# Meaning-Making Coping Methods among Bereaved Parents: A Pilot Survey Study in Sweden

**DOI:** 10.3390/bs11100131

**Published:** 2021-09-24

**Authors:** Fereshteh Ahmadi, Saeid Zandi

**Affiliations:** Department of Social Work and Criminology, Faculty of Health and Occupational Studies, University of Gävle, 80176 Gävle, Sweden; faw@hig.se

**Keywords:** bereaved families, bereavement care, coping strategies, culture, death, existential coping methods, grief, grieving parents, mourning, religious coping

## Abstract

The death of a child may result in traumatizing forms of grief, and meaning-making coping with loss seems to be important in prevention of intense psychosocial problems among bereaved parents. The aim of this quantitative pilot study was to discover the divergent meaning-making coping methods used by bereaved parents in Sweden. In doing so, 162 respondents were selected using a convenience sampling method, and they responded to the modified version of RCOPE. The study revealed that the strategies *talking to others about their feelings*, *pondering the meaning of life alone*, and *being in nature for greater emotional affiliation*, i.e., what we call secular existential coping methods, have been the most used meaning-making coping methods among Swedish mourning parents. While explaining the results, we considered the respondents’ cultural background and speculated about the potential influence of cultural teachings and elements in the selection of ways of coping with bereavement. Further, we compared the results obtained with those of the two other Swedish studies conducted among people coping with cancer and COVID-19 to further discuss the impact of culture on coping with illness, loss, grief, and crisis. The study supports the idea that culture plays an essential role in the choice of coping methods.

## 1. Introduction

The death of an offspring is considered to be the cause of the most devastating, traumatizing, and intense forms of grief. According to some studies [1,2], the death of a child impacts mortality and psychosocial problems among bereaved parents more than do other types of loss. Parental bereavement research has mainly studied parents’ grief process and responses [3,4,5]. The results of these studies show that the grief process and responses are highly individualized, long lasting, intense, and very complex. This complexity is due to the impact of many social and individual factors on coping with grief. One of these factors is culture.

Scholars have tried to reexamine grief theories and modify the current research by adapting socially and culturally sensitive aspects [6]. For instance, Neimeyer [7] adjusted aspects of the Dual Process Model [8] by incorporating a constructivist mindset into the way people understand the process of grieving. Although the issue of culture and grieving parents has been taken into account in some studies [9], in these studies, the focus has hardly been on the meaning-making coping methods used by bereaved parents, especially with the aim of identifying and analyzing the impact of cultural/contextual factors on the choice of these coping methods. One of the few studies to take the role of meaning in coping with grief into consideration is the important study by Albuquerque et al. [10]. Their study included 227 couples in the Netherlands, but cultural/contextual factors were not in focus. Moreover, Anderson et al., in a study among 57 American mothers bereaved by the sudden death of a child, found that the interaction of task coping and positive religious coping was associated with lower self-reported grief, supporting the differential utility of various coping styles on mothers’ grief reactions to the sudden death of a child [11]. To our knowledge, no studies have focused on meaning-making coping methods among bereaved parents, where the aim is to identify and analyze these coping methods in relation to cultural/contextual factors.

In this Swedish pilot study among beavered parents, the focus was on looking at meaning-making coping from a cultural/contextual perspective. The study is theoretically based on the results of 20 years of research on meaning-making coping with crises conducted by a research team in 10 countries [12,13,14,15,16,17,18,19,20,21,22,23,24].

The study, on the basis of which this paper is written, is a part of an international research project on the impact of the culture on meaning-making coping with crisis. The project is conducted among different groups such as cancer patients concerning coping with illness, academics concerning coping with COVID-19, and bereaved parents concerning coping with loss. The aim of the project is to find out different meaning-making coping methods and to discuss the impact of the culture on coping with these crises. In this regard, the studies are conducted in different cultural settings (10 countries). Concerning the study on coping among bereaved parents, it has been done only in Sweden. It is the case, therefore, that our cultural analysis is confined, here, only to explaining hypothetical reasons for applying/non-applying certain coping methods by our informants.

Therefore, in this paper, we discuss the results obtained from the current study among the bereaved parents in Sweden and also compare them with those of the two other Swedish studies conducted among people coping with cancer [25] and COVID-19 [24]. In our analysis, we proceed from a model [14] used in the framework of this international project.

### Conceptual Framework

In the current study, two kinds of meaning-making coping strategies are in focus: *secular existential coping*, and *religious/spiritual coping*. Coping can be defined as a process by which individuals try to understand and manage important demands in their lives [26] or as a search for meaning in periods of illness, stress, worry, and loss [27]. Coping is often understood as a multi-layered contextual phenomenon involving a number of basic skills [28]. Coping involves a meeting between the individual and the situation; it is multidimensional, multilayered, and contextual. The results of previous research [12,14,25], performed among people affected by cancer who lived in predominantly non-religious contexts, indicate that some coping methods that are hardly considered religious or spiritual are often used in these contexts. These strategies include “spiritual” connection with oneself, “sanctifying” nature, positive solitude, empathy/altruism, “spiritual” music, and meditation. When analyzing these methods, the researchers found out that they had much more to do with nature, to oneself, and to others than to something transcendent. They call them secular existential coping methods.

In the framework of the present study, religion is defined as a search for meaning that manifests itself in a traditionally sacred context. It is related to an organized belief system and practice associated with a sacred source that encompasses individual and institutional expressions, serving a variety of purposes [12]. Spirituality is difficult to define. One cannot place spirituality in a compartment, but it needs, according to Ahmadi, a working definition if there is an aim to carry out studies involving religion and spirituality [12]. In the framework of the present study, spirituality is defined as a search for connectedness with a source that is related or unrelated to God or another holy religious source. Spirituality involves efforts to consider metaphysical or transcendental aspects of life as they relate to forces, transcendence, and the like. Therefore, spirituality includes religion as well as many other beliefs and habits that are normally placed outside the sphere of religion.

The term *meaning-making coping methods* as it is used here entails the full range of religious/spiritual and secular existential coping methods. Ahmadi and Ahmadi developed a model for studying meaning-making coping from a cultural/contextual perspective [14]. In this theoretical model, there is no relationship between secular existential coping methods and religious ones. Further, not all secular meaning-making methods are spiritual. By criticizing the dominant theoretical frameworks in the area of religious and spiritual coping, such as the RCOPE [27] or La Cour and Hvidt’s model [29], Ahmadi and Ahmadi [14] include both the religious/spiritual coping methods and the secular existential coping methods in a conceptual framework that we use in our study.

We have conducted studies in different cultural settings, including non-religious cultures. These studies have shown that there are other secular theories, such as attribution theory [30], that we can use when studying coping in societies where religion does not play an important role in construction of people’s ways of thinking. Salander [31] discusses this point when explaining Frankl’s perspective on meaning, according to which meaning is not of ‘divine,’ but of cognitive origin. Proceeding from this perspective, we can maintain that if individuals aim to avoid feelings of meaninglessness, they may find some kind of contrasting rational (meaning). This meaning can play a decisive role in restructuring their ‘worldview.’ Salander [31] explains that:

In more secular terms, the process of giving a special meaning to objects may well be encompassed by Winnicott’s (1971) intermediate area as well as attribution theory (Fölsterling, 2001). According to Winnicott and object-relational theory, people are, from early childhood to death, able to “play with reality” (Salander, 2012). The intermediate area is the mental area of human creation: in childhood in the doll’s house or sandpit, in adulthood in the area of art and culture. It is the mental space between the internal world and external reality and it is thus both subjective and objective. Being human is being in between and thus being able to elaborate with facts, especially when confronted with unexpected negative facts such as a cancer disease.

(p. 18)

Proceeding from such a view, we categorized coping methods such as nature as secular existential coping methods.

## 2. Methodology

A quantitative research design was employed to conduct this cross-sectional study.

### 2.1. Target Group and Selection

The target group for the study is people living in Sweden who have lost their child through illness, accident, or suicide. Selection criteria were as follows: Swedish citizenship, sufficient knowledge of Swedish language. Recruitment was accomplished through the help of two support groups, as explained below. The sample size was 162. The sample was certainly not representative of all beavered parents in Sweden; however, the results can provide a good indication of what the situation might look like for other parents with the same painful experience.

It should be mentioned that conducting the survey among bereaved parents was not an easy task. There are no official records of families affected by bereavement in Sweden. Therefore, we had to garner help from support groups in order to be able to recruit our participants; however, several support groups refused to cooperate with us in data gathering, despite the fact that the survey was approved by the Swedish Ethical Review Authority. The reason for their refusal is that one of the most important aims of a support group is certainly to help this very vulnerable group to cope with their grief; such a survey may, however, bring to life certain painful situations that can hinder the coping process. It is the case that every bereaved parent can themselves as an adult person decide to participate or refuse to participate in a survey and take responsibility for their participation, but when participants are *recruited through* a support group, the support group is also responsible for the consequences. 

### 2.2. Primary Data Collection

Data collection was accomplished using a web survey. Due to nature of the population and the possibilities at hand, we did not attempt to secure a quota or use random sampling. Instead, using a convenience sampling method, we distributed the questionnaire through the help of two support groups for bereaved parents. The web address of the electronic survey, which was directly connected to the Sunet Survey at the University of Gävle, was published on the media page of these support groups, which have members throughout Sweden.

### 2.3. Secondary Data

Searching the well-known scientific databases for studies concerning meaning-making coping methods in Sweden, we found only two articles, both of which belong to our own international project. These studies include one study concerning Swedish patients coping with cancer [25], and another study concerning Swedish academics coping with COVID-19 [24]. Since we found no other studies with a similar instrument than these two studies, while mentioning the comparison work, we have referred merely to these two studies throughout this paper.

### 2.4. Measure

The questionnaire was partly based on a modified version of RCOPE (covering meaning, control, comfort/spirituality, intimacy/spirituality, and life transformation) [32], and the results were partly obtained from our other studies (quantitative and qualitative) conducted in Sweden for inquiring into the applied meaning-making coping. We also added very few new coping methods based on the crisis in focus in the present study, i.e., the loss of a child. These new coping methods are found in the interviews, which were conducted before modifying the questionnaire. This modified version of RCOPE includes 26 Swedish items rated on a five-point Likert scales ranging from 1 (“never”) to 5 (“always”); each of these 26 items refers to one of the meaning-making coping methods. An additional 12 background items were also included in the questionnaire. To be sure that the questionnaire has a common attitude behind, which means high correlation between the questions used, we calculated Cronbach’s alpha. The reliability test showed that this set of questions had a value of 0.86, which is a very high reliability (>0.7). The main instrument was validated for form, language, and content in earlier studies [24,25]. Using Cronbach’s alpha method, we found that these studies also reported a very high reliability coefficient for this questionnaire.

### 2.5. Data Analysis Methods

The use of 26 coping methods was examined. Throughout the present article, we present how the respondents answered the various questions in the survey as well as a number of frequency charts. A correlational analysis was conducted between how often one has used a method and how well one has managed to deal with one’s grief. This is to demonstrate which methods have had the greatest effect on the respondents’ grief work. The data were analyzed using SPSS Statistics 27 (IBM^®^, Chicago, IL, USA).

### 2.6. Description of the Respondents

**Figure 1:**
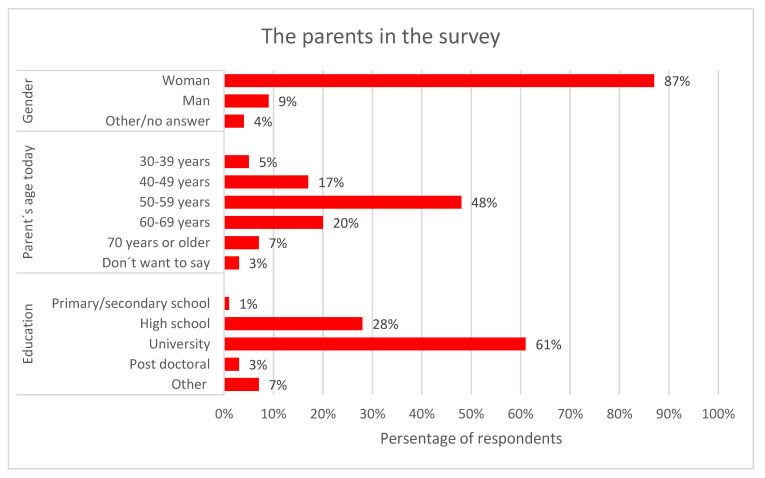
Respondent demographics.

Nearly 90% of respondents were the mother of the lost child. In terms of age, 48% were in the 50–59 age range. The majority of respondents had a university or college education (Table 1 indicate the overrepresentation of women, age 50–59 years, and university education in our cohort). The data on the parents who participated in the study are presented in Figure 1. 

**Figure 2:**
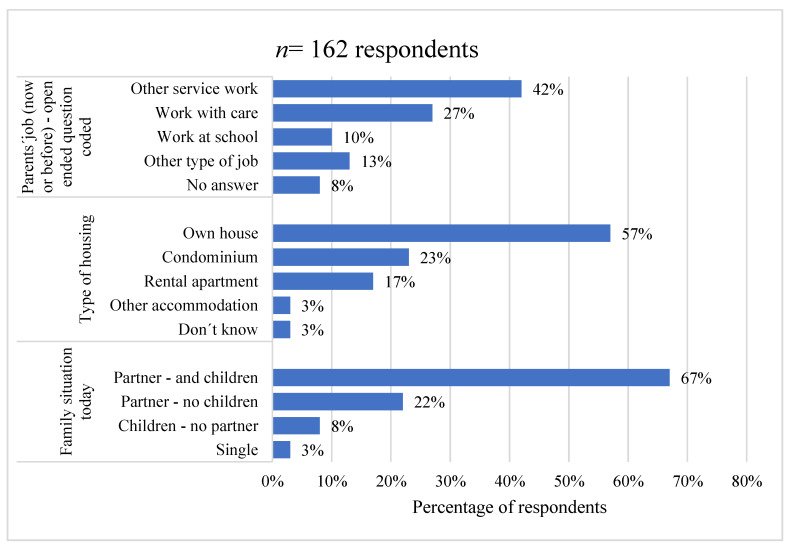
Respondents’ life situation.

The question of parents’ occupation is an open question and has been coded. The study shows that most people worked, or have worked if they are pensioners, as a (private) official. The officials who stated that they had some type of service in the schools, health, and social care were broken out and reported on separately. As Figure 2 shows, the majority (57%) lived in villas, townhouses, or semi-detached houses, while almost a quarter (23%) lived in condominiums.

Concerning the family status of informants, 67% had partners and children (domiciled or emigrated); while 22% lost their only child but had a partner; 8% had children but no partner; and 3% were single, i.e., they had neither any children nor partner.

One parent stated in the open answers that she had lost two children. The respondents lived in over 50 different Swedish cities. Roughly estimated, about 10% lived in the Stockholm area. About 90% were themselves raised in Sweden, while the others were raised in another European country.

**Figure 3:**
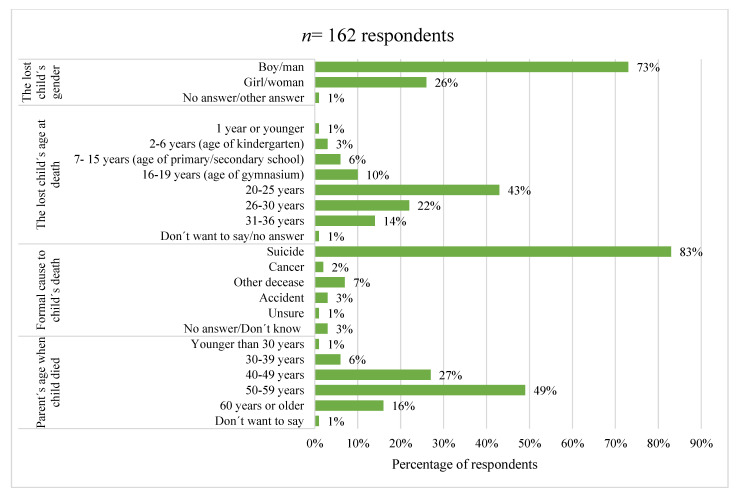
Demographics of the deceased child.

As Figure 3 reveals, the child these parents reported having lost was often a boy or man, more precisely in 73% of cases. At the time of death, almost half (43%) of the lost children were in the age range of 20–25 years. Moreover, 20% were younger than 20 years and 36% older than 25 years. The proportion of deceased children increased with increasing age groups up to 20–25 years, where it culminated, and thereafter the proportion decreased gradually. The oldest child was 36 years, while the youngest had not turned one year. Half (49%) of the parents who participated in the study were between the ages of 50 and 59 at the time of their child’s death. As many as 76% were in the age range of 40–59 years when their child passed away.

Almost 28% of respondents claimed to be believers, while relatively few considered themselves unable to know adequately whether God (or the equivalent in any other religion) exists, i.e., they were agnostics (16%) or did not at all believe in a god, that is, pronounced atheists (14%). However, most people (42%) stated that they had no belief at all or that they were uncertain in general and expressed several opinions.

### 2.7. Ethical Considerations

The study was conducted in accordance with the Declaration of Helsinki. The survey was approved by Swedish Ethical Review Authority (Dr. 2019-01641). In a short letter attached to the survey, the respondents were informed about the study, their possibility to withdraw, and the use and preservation of data. The respondents were also informed that they gave their consent as they responded to the survey.

## 3. Results

It is notable that since there is not any official records of parents who have lost their child in Sweden and since the size of sample is limited, we cannot maintain that our sample is representative of the totality of the bereaved parents in Sweden. Our results cannot therefore be generalized to all these parents in Sweden.

### 3.1. The Most Common Coping Methods

**Figure 4:**
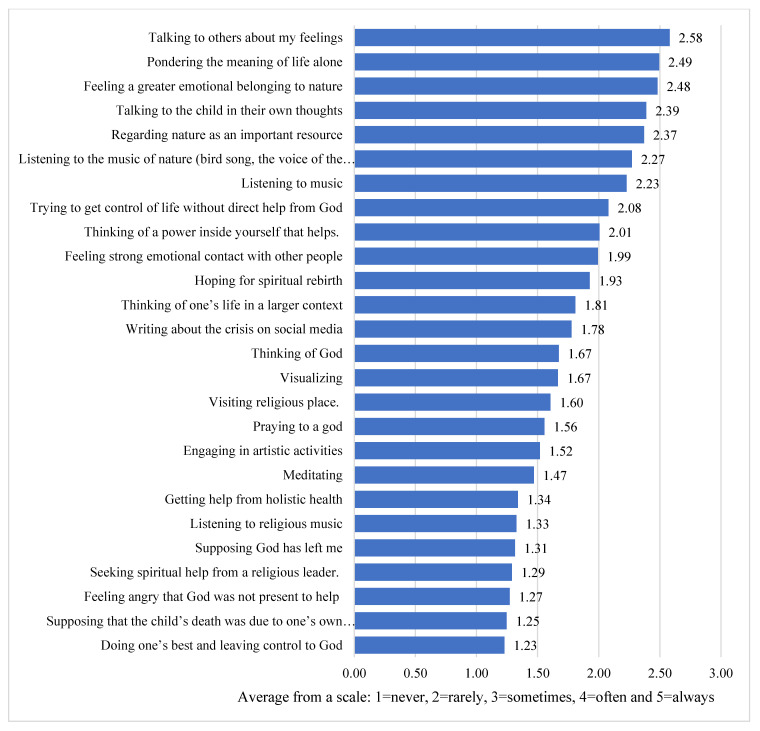
Frequency of coping methods used.

Figure 4 shows which coping methods have been most common among respondents.

The figure shows that the most common method was *talking to others about their feelings*, followed by *pondering the meaning of life alone* and *being in nature for greater emotional affiliation*, i.e., what we call secular existential coping. Below is a more detailed analysis of how frequently different coping methods have been used, but also which subgroups have used the respective methods to manage the crisis after their child’s death. The coping methods are reported in chronological order according to how frequently they have been used.

**1. Talking to others about my feelings.** The highest average (2.58) was found for the factor *talking to others about my feelings*. Almost 19% of respondents stated that they *always* did this, and another 49% *often*, which means that as many as 68% chose *often* or *always* the method *talking to others about my feelings*. Only 3% stated that they *never* do this. It was mainly the subgroup ‘believers’ who reported using the coping method *talking to others about my feelings* (2.8), where as many as 80% said they have *always* or *often* done this. Besides faith, having lost a daughter is another factor that seems to have contributed to the choice of this coping method. Other subgroups that have chosen the alternative *often* for this method, more than other subgroups did, were women, respondents younger than 60 years, those with a university education, those whose child was 20–25 years at death, as well as those aged 50–59 at the time of their child’s death and those who had not grown up in a religious family.

**2. Pondering the meaning of life alone**. The second most common way of dealing with this crisis, seen as the next highest average (2.49), was *being alone and reflecting on the meaning of life*. One-fourth (26%) stated that they *always* did this and another 35% that they *often* did this. Again, only 3% reported *never* doing this. It was mainly respondents in the 50–59 age group who mentioned this way of dealing with their crisis (mean 2.72). Losing a daughter also seemed to have contributed to doing this more *often*. Subgroups that did this more often than other subgroups were women, particularly those younger than 60 years with a university education, those whose child’s age was 20–25 years at death, those who were 50–59 years at the time of the child’s death, and those who were not raised in a religious family.

**3. Feeling a greater emotional belonging to nature.** The third most common way of dealing with this crisis, seen as the third highest average (2.48), was *feeling a greater emotional belonging to nature*. Almost 20% reported *always* and 37% reported *often* utilizing this coping method. Only 5% reported *never* having used this method. It was primarily those who grew up in a religious family who reported using this method (mean 2.67). Women, those 50–59 years, those with an upper secondary education, and those who are agnostic today also reported having used this method relatively *often*. The child was usually a daughter and the child was between 20 and 25 years. Further, those whose child was a daughter and those whose child was 20–25 years belonged to the group who reported using this method *often*. 

**4. Talking to the child in their own thoughts**. The fourth most common way of dealing with this crisis was *talking to your deceased child in your own thoughts*. The alternative *always* was chosen by 35% and the alternative *often* by 25%. About 12% reported *never* having done this. It was mainly those with the highest secondary education and those who stated that they were an agnostic who reported this. Women and those in the 50–59 age group reported having used this coping method more than other groups. In addition, those whose child was a daughter and those whose child was 20–25 years reported using this method *often*. 

**5. Regarding nature as an important resource.** The fifth most common way of dealing with this crisis, seen as the fifth highest average (2.37), was *regarding nature as an important resource* in dealing with the grief. A total of 28% reported that they *always* and 29% that they *often* used nature as a coping method, i.e., 57% used nature *always* or *often* as an important resource in coping. Again, 10% reported *never* having used this method. Those who grew up in a religious family and those who were currently agnostics belonged to the group who *often* use this coping method. Women and the oldest age group, i.e., >60, also used this method *often*. Moreover, those whose child was older than 26 at death used this method *often.*

**6. Listening to the music of nature**. This was the sixth most common coping method used by our respondents. Half of them (50%) listened to the music of nature *often* or *always*. Almost 16% reported *never* having done this. This method was most *often* used if the child was a daughter and/or older than 26 years at death. It was also relatively common if parents were older than 60 and/or agnostic. Again, this was done more *often* if the child died by suicide.

**7. Listening to music**. This coping method was the seventh most common way of dealing with crisis. Almost half (47%) used it *often* (36%) or *always* (11%). Few (6%) *never* did this. As the study shows, this coping method was most common among believers and among those whose child was 20–25 years at death. Parents who used this method were often older than 60. This was otherwise one of the few methods used more often by fathers than by mothers.

**8. Trying to get control of life without direct help from God**. The eighth most common method of dealing with this crisis was *trying to get control of life without direct help from God*. Almost half (45%) (*often* 22% and *always* 23%) of respondents reported using this coping method. A relatively large proportion (28%) stated that they had never done this. It was most common among atheists. Otherwise, this was another one of the few methods used more often by fathers than mothers. These respondents more likely were older than 60 and had a university education. Again, this method was used more often if the child died by suicide.

**9. Thinking of a power inside yourself that helps**. This coping method was the ninth most common way of dealing with crisis. A total of 36% reported having *often* or *always* thought in this way, whereas 17% reported *always* doing so. Moreover, 19% stated that they never thought of this power. This method was most common among respondents whose child died in a manner other than suicide, as well as among those who claimed to be believers and were raised in a religious family. It was much more common among women than among men, and also more common among those with a lower education as well as those whose child was older than 26 years at death.

**10. Feeling strong emotional contact with other people**. The 10th most common method of dealing with one’s crisis was *feeling strong emotional contact with other people*. A total of 32% have had this kind of contact *often* or *always*, but only 6% said this was the case *always*. However, the same proportion reported *feeling emotional contact with other people* as their coping method *sometimes* (32%), and only 11% reported *never*. It is the believers and respondents who grew up in a religious family who typically had this emotional contact with other people. Otherwise, women used this coping method more often than men. This was also the case for respondents in the age range of 50–59, those with lower education, and those whose child was 20–25 years at death, as well as those who were 50–59 at the time of the child’s death.

**11. Hoping for spiritual rebirth.** In 11th place was coping in the form of *hoping for spiritual rebirth* (mean 1.93). About 19% *always* hoped for this, and another 19% *often* did, but on the other hand, relatively many (27%) reported *never* hoping for spiritual rebirth. It was above all the younger group under 50 who have hoped for spiritual rebirth. People in this group also belonged to the younger group (≤49 years) when their children passed away, and their children were 20–25 years at death. They also tended to be believers and to have grown up in a religious family. Women reported this belief more often than men did.

**12. Thinking of one’s life in a larger context**. The 12th most common way of dealing with the crisis was *thinking of one’s life in a larger context* (mean 1.81). Almost 14% thought in this way *always*, and 15% *often*. Here, too, a larger group (27%) reported *never* thinking this way. It was mainly the believers and those who grew up in a religious family who reported *often* having these thoughts. It was also relatively common that the cause of death was not suicide. These respondents were also more often women and younger parents, and those belonging to the group of parents who were younger (≤49 years) at the time of the child’s death.

**13. Writing about the crisis on social media**. The 13th most common way to manage one’s crisis was to write about it on social media (average 1.78). Slightly more than 28% reported doing this *always* or at least *often*, but more (34%) reported *never* having done this to deal with their crisis. It was mainly younger parents—under 50—who used this coping method. This strategy was also more common among believers and those who grew up in a religious family. Parents in this group were more likely to have had a son who had not committed suicide. They were also more often women and younger and tended to belong to the group of parents who were younger (≤49 years) at the child’s death.

**14. Thinking of God.** The 14th most common way of managing one’s crisis was *thinking of God* (mean 1.67), with 10% *always* doing so and another 16% doing so *often*. A very large group (38%) reported *never* having thought of God to deal with their crisis. It was above all the believers and those who grew up in a religious family who most often had these thoughts. In this case, it was more common for women to think of God as a method of coping with the crisis.

**15. Visualizing**. The 15th most common way of dealing with the crisis was *visualizing* (average 1.67). Only 6% reported *always* visualizing as a coping method, but another 11% had *often* done it. Here, a very large group (34%) reported *never* visualizing in order to deal with their crisis. It was mainly the believers and those who grew up in a religious family who typically did this. Those who had a son who died without committing suicide *often* used this method. Even in this case, women were more likely to have done this than men.

**16. Visiting religious place.** The 16th most common coping method of the 26 methods was *visiting religious places* (average 1.6). Only 5% had used this method *always*, but another 11% had done so *often*. Here, a very large group (34%) reported never having gone to a religious place. It was mainly the believers and those who grew up in a religious family who tended to use this method. In this case, it was more common among women than among men.

**17. Praying to a god.** Number 17 of the 26 methods of dealing with one’s crisis involves a religious act, namely, *praying to a god* (mean 1.56). Some did this *always* (10%), and just as many did it *often* (10%). Here, too, a very large group (44%) responded that they had never prayed to a god. This method was mainly used by the believers and those who grew up in a religious family. In this case as well, more women than men used this method.

**18. Engaging in artistic activities**. *Engaging in artistic activities* to manage the crisis was in 18th place (average 1.52). Some respondents did this *always* (7%), and slightly more did so *often* (11%). However, it was most common to *never* use this coping method (46%). Above all, this method was most common in the group who lost a child to something other than suicide. Even those who grew up in a religious family did this relatively often. This method was relatively common among men and women, although women used it somewhat more often. It was also more common among older parents, those whose child did not die very young, and those who were older when their child passed away.

**19. Meditating**. In position 19, we find *Meditating* as a coping method. Very few people *always* did this (3%), but some did it *often* (12%). A majority of respondents reported *never* having used this method (56%). Above all, it was most common in the group that lost a child to something other than suicide, but also relatively common among the believers and those who grew up in a religious family. This method, too, was more common among women than among men, and slightly more common among older parents and those with a university education.

**20. Getting help from holistic health**. The coping method of *getting help from holistic health* (mean 1.34) was in 20th place. Very few respondents reported doing this *always* (3%), but some used this method *often* (6%). However, a clear majority of respondents reported *never* having done this (63%). Above all, it was most common in the group of parents who lost a daughter and who themselves were relatively young when it happened. It was also more common if the child did not commit suicide, and relatively common among the believers and those who grew up in a religious family. It was more common among women than among men.

**21. Listening to religious music**. The 21st most common coping method for dealing with the crisis was again a religious act, namely, *listening to religious music* (mean 1.33). Only 2% reported using this method *always*, while slightly more (6%) did so *often*. A very large group (60%) reported *never* having listened to religious music. It was mainly the believers and those who grew up in a religious family who typically used this coping method. In this case, too, use of this coping method was more common among women than among men, among those who were 60 years or older, and among those with a college education.

**22. Supposing God has left me**. In place number 22 of 26, we found the coping method *supposing God has left me* (mean 1.31). Almost 10% had this idea *often* or *always*. Here, a large group (74%) reported *never* thinking this. It was mainly the younger group (≤49 years) and atheists who thought in this way. Women thought this more often than men.

**23. Seeking spiritual help from a religious leader**. As the most common way of dealing with one’s crisis, *seeking spiritual help from a religious leader* (mean 1.29) was in 23rd place. Only 4% reported having done this *always*, while 3% said they did this *often*. Here, too, a very large group (66%) responded that they had *never* sought spiritual help from a leader. It was mainly the believers who used this method, as well as those who grew up in a religious family. In this case, too, it was more common for women to do this compared to men.

**24. Feeling angry that God was not present to help.** *Feeling angry that God was not present to help* (mean 1.27) was the 24th coping method on the list of 26 coping methods. Almost 10% had this feeling *often* or *always*. Here, too, a very large group (74%) reported *never* having considered this. It was mainly agnostics and those who belonged to the younger parent group (≤49) who felt this way. In this case, too, use of this coping method was more common among women.

**25. Supposing that the child’s death was due to one’s own actions/not being a strong enough believer**. At 25th place came the idea of losing the child due to one’s own actions or for not believing enough (mean 1.25). Almost 9% had thought this *often* or *always*. Here, too, a very large group (81%) reported *never* having thought in this way. It was above all the younger group of parents, whether current age or age at the child’s death, who considered this. Those whose child died younger were more likely to have thought in this way. Here, too, women used this coping method more often than men.

**26. Doing one’s best and leaving control to God**. In last place on our list of coping methods, we found *doing one’s best and leaving control to God* (mean 1.23). Only 6% did this *always* or *often*. Here, too, a very large group (75%) reported *never* having used this method. Mainly believers used it, but it was also relatively common among those who grew up in a religious family. Those whose child died young considered this more often than those whose child was older. Even in this case, this method was more common among women than among men, as well as among those with a university education.

In Table 2, these 26 coping methods are classified into two categories according to the meaning-making coping theory and the employed questionnaire’s components: *religious/spiritual coping methods (RCOPE)* and *secular existential coping methods*. The number in front of each method shows its ranking by frequency of use.

As the results show, religious coping methods were the lowest in the ranking of all reported coping methods. The most prevalent coping methods were clearly the secular existential ones. This confirmed the findings obtained in previous studies on meaning-making coping among cancer patients in Sweden [13,14].

### 3.2. Effective Coping

The findings indicate that, regardless of the coping method(s) used, more than 64% of respondents believed they have succeeded rather or very well with their crisis management. Only 9% thought coping worked rather or very poorly. The groups that considered themselves to have handled the crisis best were those in the 50–59 age group, where 23% managed the crisis very well and 68% rather or very well. Other subgroups that managed the crisis relatively well (all with at least 2.6 on average) were those with a university education, those who lost a son, those whose child was older than 26 years, and those whose child did not commit suicide. Those who were believers and agnostics, respectively, considered themselves to have handled the crisis better than those who were atheists or uncertain in their view of life.

**Figure 5:**
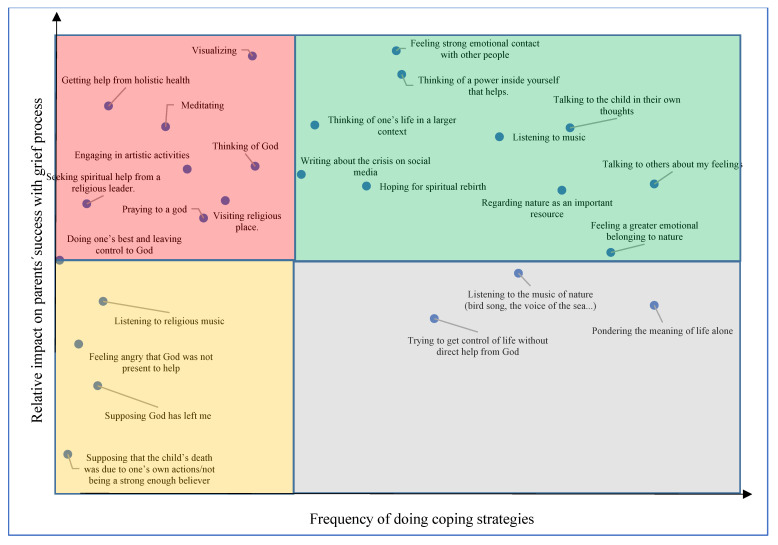
Frequency of different coping strategies and their impact on perceived success in grieving.

How often one uses a coping method is not equivalent to its perceived effectiveness in managing one’s crisis. In Figure 5, all coping methods above the horizontal line show a positive correlation between frequency of use and perceived effectiveness in coping with grief. For the methods below the line, this correlation was negative. The coping method with the greatest impact on perceived success in grieving was *thinking of a power inside yourself that helps*. That this method lies in the GREEN square means that relatively many used this method relatively often. The method that has the strongest correlation with perceived success in grieving was *feeling strong emotional contact with other people*. The methods found in the RED quadrant showed a relatively strong impact on perceived success in grieving, but that relatively few used these methods today. Along with the method of being able to practice *visualizing*, the analysis showed that it had a relatively strong impact on perceived success in grieving. The coping methods that fell to the left below the horizontal line (YELLOW square) are those few made use of and that had a relatively small effect on whether or not they coped well with their grief. In the GRAY square on the right are the methods that many people use, but that had a relatively small effect on perceived success in grieving.

## 4. Discussion

This study made attempts to identify the ways in which bereaved parents in Sweden cope with loss of their child. The study revealed that *talking to others about their feelings*, *pondering the meaning of life alone*, and *being in nature for greater emotional affiliation*, i.e., what we call secular existential coping methods, have been the most used meaning-making coping methods. Below, we discuss the findings, bringing forth some aspects of the Swedish culture.

### 4.1. Cultural Perspective

There are studies indicating that religion has helped believers decrease their level of depression after a loss [33] or shorten the period of recovery after a loss [34]. According to Halifax, religiosity is a positive factor for helping the bereaved find solace and acceptance [35]. However, as Mohamed Hussin et al. [36] maintains:

The question of how religion helps bereaved parents and to what degree religion helps bereaved parents cope with their loss are still scarcely explored. Despite the discussion of the positive roles of being religious in bereavement, there are also debates that claim religious faith may cause negative impacts on bereaved parents.

(p. 2)

One of these negative impacts is that religious faith can bring about the idea that the loss of one’s child is a punishment, and such an understanding can negatively affect coping with bereavement [37]. The religious coping method *supposing that the child’s death was due to one’s own actions/not being a strong enough believer—*where loss of the child is seen as a punishment—was 25th in frequency among the total of 26 coping methods. Only 9% of respondents reported having had this thought *often* or *always,* with a very large group (81%) reporting *never*. The reason can be found in the strong dominance of secular culture in Swedish society and the fact that religion does not play an important role for the majority of the Swedish people. As the latest World Value Survey shows, almost 75% of Swedish people believe that religion is not very important or not at all important in their life [38]. A brief study of Swedish history also indicates that belief in a personal God has decreased in Sweden during past decades, whereas belief in a transcendent power has increased [39].

This explains also why despite the fact that the coping method “talking to others about my feelings” is one the most prevalent coping methods among the informants, the religious coping method “seeking help from a religious leader” is among the least used coping methods. It is not then difficult to realize that these “others” are not religious leaders. It is, therefore, the coping method “talking to others about my feelings” that is not regarded as a religious coping method, but a secular one, even when the religious people use it and even though congregational life is usually regarded as a mainstay of the three Abrahamic religions. Proceeding from a cultural perspective, we can explain that Swedes do not often turn to church/religious leaders in time of crisis. Church does not have the strong power that it has in the south of Europe or in the USA.

Regarding religious coping, one interpretation of the present and other results on meaning-making coping in Sweden [12,13,14] is that those few respondents who report using these coping methods relied not only on God’s power but on their own as well. They felt they were responsible for their own life and viewed God as a partner who helped them solve their problems. Here, God could not pull person out of a difficult situation but could extent a hand to help the person get through it. The respondents did not wait around passively for help from God. Thus, although God was in view and could help the individual manage the situation, the individual was in charge, not God. There was a clear tendency toward control and self-direction attributes that are strong among Swedes. It is perhaps why, in the present study, the religious coping method *doing one’s best and leaving control to God* is the least frequently used coping method.

Another interesting point concerning the impact of cultural context on coping in the present study is the coping method for bereavement that involves parents’ continued bonding with the deceased child. Here, religion is considered, as Mohamed Hussin et al. [36] write, “a tool that reconnected the deceased child with the bereaved parents” (p. 8). They emphasize the following in their study:

Religious activities such as prayers, donations, or performing the Hajj (an annual pilgrimage to Mecca) for the deceased child were described as a “bridge” that signified the remembrance of the deceased child. In addition, the bereaved parents believed that religion taught them that even after death, they were still able to give reward to their deceased child.

In our study, the issue of continued bonding with the deceased child did not occur within the framework of religious activities but through the secular existential meaning-making coping method *talking to the child in their own thoughts*. This coping method was the fourth most common coping method used by the bereaved parents in the present study. The alternative *always* was chosen by 35% and the alternative *often* by 25%.

### 4.2. Comparison

The study indicates that the secular existential meaning-making coping methods are the most used meaning-making methods by bereaved parents. In the following, by comparing the results of our study with the one conducted in the framework of the project “Meaning-Making Coping with Cancer,” [25] and the other conducted under the project “Coping methods in Sweden during COVID-19 crisis,” [24] we tried to shed more light on the certain results obtained in our study among grieving parents. All these three studies have been conducted among people in Sweden. Several points are found when comparing these three survey studies (one here is referred to as Coping Cancer, another as Coping COVID, and the other as Coping Grief) that are interesting and worthy of further investigation.

- The general pattern found in Coping Grief was the same as that found in both Coping Cancer and Coping COVID: the most frequently used coping methods were secular existential coping methods and the least frequently used ones were religious/spiritual methods.
- In Coping Cancer, praying was the 13th method in the ranking of the 24 methods. In Coping Grief, it was ranked lower, at 17 of 26. In Coping COVID, praying was the seventh method out of 15 methods. In all three studies, praying was not among the most frequently used methods, but the different places they occupied in ranking was interesting. In Coping Grief, praying was ranked lower than that in Coping Cancer. The reason may have been that cancer patients believed praying could help change the situation, i.e., being cured, while in case of bereaved parents, changing the situation was impossible as the child was already dead. The partially higher share of praying in Coping COVID, compared to the other two studies, may have been due to the unknown nature of the virus and the unprecedented situation caused by COVID-19, directing some people to ask for help from the realm of the divine.
- Although the three studies showed that connection with others played the role of a coping method, in Coping Grief, this method was used by more respondents. This maybe because, for bereaved parents, confabulating with and confiding in others were mainly for emotional discharge, memorialization, and remembrance; for trying to express negative emotions; and also to keep the memory of the lost child alive [40,41]. In contrast, in Coping Cancer and Coping COVID, the respondents were more inclined to want to forget the crisis.
- All the three studies showed that nature was an important coping method. However, in Coping Cancer and Coping COVID, more participants used this method than in Coping Grief, perhaps because illness and health are more related to nature.

## 5. Conclusions

The current study provides evidence that the most used coping methods are those that we call secular existential coping methods, as it was the case in the two other studies mentioned above. We attempted to explain the findings by taking into consideration the aspects of culture. The study identifies the existential coping strategies more often employed by mourning parents in Sweden; it also elucidates the potential role of secular existential meaning-making coping methods in facilitating parental bereavement at the death of a child and thereafter introducing some possible cultural traces of these strategies. Our study may aid in enriching the research on understanding the human encounter with death by disclosing how Swedish parents deal with this crisis. 

We hope to contribute to an increase in scientific knowledge in the death studies and grief field and to allow organizations, practitioners, and scholars interested in death, dying, mourning, bereavement, and grief support to plan evidence-based, culturally tailored strategies to facilitate the grief process for families. We recommend that loss psychologists and grief counselors and therapists, avoiding the application of a one-size-fits-all model of grief and considering the cultural and religious diversities of the communities and clients, make use of the present findings when providing advice to grieving spouses. Acknowledging and understanding the cultural variations in grieving for loss experiences and the related narratives and meaning makings are of high importance, as published research also indicates that people in different cultures react differently to loss [42] and a grieving parent culture may offer support in a search for meaning [43]. More comparative, cross-cultural, and ethnographically informed investigations, as well as in-depth qualitative inquiries, are needed to refine what is known and to explore what is not known in the field.

### 5.1. Limitations

Our study also has its limitations. There may be a potential for bias in the sample that was recruited via support groups. Moreover, statistical studies are useful for generalization purposes; however, to catch the experiences and processes of the meaning-making coping with bereavement and loss, in-depth interviews and longitudinal inquiries among parents suffering the death of an offspring in very different moments of their grief would be more informative.

### 5.2. Practical Implications and Policy Recommendations

- Better knowledge of various coping methods involving meaning-making may help social work and psychological care efforts to better help bereaved families manage the effects of loss and grief.
- In countries where religion is integral to people’s lives, planners should pay considerable attention to religious/spiritual coping methods. However, as well as facilitating religious coping methods for those experiencing psychological distress stemming from crises such as loss of a child, these planners should also allot funds and facilities for promoting existential coping methods.

## Figures and Tables

**Table 1:** Composition of cohort and structure of the general Swedish population.

		Present Study	Swedish Population 2019 (SCB)
**Gender**	Women	87%	49%
	Men	9%	51%
	Other/no answer	4%	-
**Age groups** **(share of interval 30+ years)**	30–39 years	5%	22%
	40–49 years	17%	20%
	50–59 years	48%	20%
	60–69 years	20%	17%
	70 years or older	7%	21%
	Unknown	3%	-
**Education**	Elementary school	1%	11%
	High school	28%	43%
	University	61%	46%
	Other	7%	-

**Table 2:** Religious/spiritual coping methods vs. secular existential coping methods.

Secular Existential Methods	Religious/Spiritual Methods
1. Talking to others about my feelings 2. Pondering on the meaning of life alone 3. Walking in nature and feeling great emotional belonging to nature 4. Talking to the child in their own thoughts 5. Regarding nature as an important resource 6. Listening to the music of nature (bird song, voice of the sea, etc.) 7. Listening to music 8. Trying to get control of life without direct help from God 9. Thinking of a power inside yourself that helps 10. Feeling strong emotional contact with other people 12. Thinking of one’s life in a larger context 13. Writing about the crisis on social media 15. Visualizing 18. Engaging in artistic activities 19. Meditating 20. Getting help from holistic health	11. Hoping for spiritual rebirth 14. Thinking of God 16. Visiting the religious places 17. Praying to a god 21. Listening to religious music 22. Supposing God has left them 23. Seeking spiritual help from a religious leader 24. Feeling angry that God was not present to help 25. Supposing that losing of one’s child was because of one’s actions/not being a strong enough believer 26. Doing one’s best and leaving control to God

Note: The number in front of each method shows its ranking by frequency of use.

## Data Availability

The datasets presented in this article are not readily available because by the time of the data collection, participants were not informed about the possibility of making data available to outside researchers. Requests to access the datasets should be directed to Fereshteh Ahmadi, Fereshteh.Ahmadi@hig.se.
